# Patterns of lithium exposure and mortality in bipolar disorder: A population-based cohort study

**DOI:** 10.1192/j.eurpsy.2025.10090

**Published:** 2025-08-27

**Authors:** Vincenzo Oliva, Michele De Prisco, Gerard Anmella, Clàudia Valenzuela-Pascual, Ariadna Mas, Tábatha Fernández, Giovanna Fico, Andrea Murru, Marc Valentí, Jordi Blanch, Joaquim Radua, Allan H. Young, Eduard Vieta, Diego Hidalgo-Mazzei

**Affiliations:** 1 Bipolar and Depressive Disorders Unit, Department of Psychiatry and Psychology, Hospital Clínic de Barcelona, Barcelona, Catalonia, Spain; 2 Institut d’Investigacions Biomèdiques August Pi I Sunyer (IDIBAPS), Hospital Clínic de Barcelona, Barcelona, Catalonia, Spain; 3 Department of Medicine, School of Medicine and Health Sciences, Institute of Neurosciences (UBNeuro), University of Barcelona (UB), Barcelona, Catalonia, Spain; 4 Biomedical Research Networking Centre Consortium on Mental Health (CIBERSAM), Instituto de Salud Carlos III, Madrid, Spain; 5 Centre for Affective Disorders (CfAD), Institute of Psychiatry, Psychology and Neuroscience (IoPPN), King’s College London, London, UK

**Keywords:** bipolar disorder, lithium, mortality, population-based cohort, treatment continuity

## Abstract

**Background:**

Lithium treatment is associated with reduced mortality in bipolar disorder (BD), but the role of treatment continuity remains underexplored. This study investigated the association between patterns of lithium exposure and all-cause mortality in a population-based cohort.

**Methods:**

We analyzed electronic health records from 15,384 individuals with BD in Catalonia, Spain (2010–2019). Patients were classified as having sustained, partial/intermittent, or no lithium exposure based on annual defined daily doses (DDDs). All-cause mortality was the primary outcome. Kaplan–Meier and Cox regression analyses (adjusted for sociodemographic, clinical, and treatment-related variables) estimated hazard ratios (HRs) for mortality risk. Interaction and sensitivity analyses were conducted to assess the role of comorbidity burden and dose effects.

**Results:**

Over the study period, 715 deaths were recorded. In fully adjusted models, sustained lithium exposure was associated with a significantly lower mortality risk compared to no exposure (HR = 0.69, 95% confidence interval [CI]: 0.51–0.93, p = 0.016). In the lithium-exposed subgroup, sustained use was also protective compared to partial/intermittent exposure (HR = 0.70, 95% CI: 0.51–0.97, p = 0.03). No significant interaction was observed between sustained lithium use and comorbidity burden. Sensitivity analyses confirmed this effect at lower dose thresholds but not at higher ones.

**Conclusions:**

Sustained lithium use is associated with improved survival in BD. Discontinuous exposure does not confer the same benefit. Ensuring treatment continuity may maximize lithium’s protective effect and improve long-term outcomes.

## Introduction

Bipolar disorders (BD) are severe mental illnesses characterized by alternating episodes of (hypo)mania and depression, affecting approximately 1% of the global population [1]. These conditions are associated with significant functional and markedly elevated mortality risk, about 2.6 times higher than the general population, with an average reduction in life expectancy of 7 years [2, 3]. Both unnatural causes (e.g., suicide) and natural causes (e.g., cardiovascular and metabolic comorbidities) contribute to this mortality gap, highlighting the need for effective long-term management strategies that address both mood symptoms and survival [4].

Lithium, a cornerstone in BD treatment, is well known for its mood-stabilizing and anti-suicidal properties [5]. Beyond these effects, emerging evidence suggests a broader protective role in reducing overall mortality. Meta-analyses of randomized controlled trials and longitudinal studies have identified lithium as the mood stabilizer associated with the lowest all-cause mortality risk [6–8], including in first-episode and severe BD cases [9, 10].

Some studies have also suggested that longer lithium treatment duration and higher cumulative exposure are associated with lower mortality risk [8]. However, most studies have classified patients simply as lithium-exposed or unexposed, without considering treatment continuity. Discontinuation is linked to increased relapse [11] and suicidality [12], but its impact on mortality remains understudied. One large nationwide study found higher mortality among patients who discontinued lithium compared to those who discontinued valproate, suggesting that lithium discontinuation may be especially detrimental [13].

To address this gap, we examined the association between lithium exposure patterns and all-cause mortality in a large population-based cohort of individuals with BD in Catalonia, Spain. By distinguishing sustained, partial or intermittent, and no exposure, we aimed to clarify the role of treatment continuity in survival, extending our understanding of lithium’s protective effects beyond suicide prevention to long-term mortality outcomes.

## Materials and methods

### Study design and population

This prospective cohort study utilized data from the “Programa d’analítica de dades per a la recerca i la innovació en salut” (“Data Analytics Program for Health Research and Innovation” [PADRIS]), managed by the Agency for Health Quality and Assessment of Catalonia (AQuAS). PADRIS integrates anonymized sociodemographic, clinical, and healthcare data from the public health system in Catalonia (CatSalut), in line with legal, ethical, and transparency standards [14]. Since most of the Catalan population uses public healthcare, PADRIS provides a highly representative sample. All data are fully anonymized, with no possibility of patient identification. The study was approved by the Hospital Clínic of Barcelona Ethics Committee (HCB/2020/0735).

The PADRIS dataset included 473,812 individuals who accessed public specialized mental health services (outpatient, inpatient, or emergency) between January 1, 2015, and December 31, 2019. Retrospective follow-up extended back to January 1, 2010, using data from electronic health records for the entire period. Information was anonymized and de-identified in full compliance with legal and ethical standards. Additional details are reported elsewhere [15–17].

This study followed the Strengthening the Reporting of Observational Studies in Epidemiology (STROBE) guidelines for reporting observational research [18].

### Exposure and outcomes

We included patients diagnosed with BD according to the International Statistical Classification of Diseases and Related Health Problems 10th Revision (ICD-10). Based on lithium exposure, patients were categorized into three groups: sustained lithium exposure, partial or intermittent lithium exposure, and no lithium exposure. Lithium exposure was measured in defined daily doses (DDDs), as established by the WHO Collaborating Center for Drug Statistics Methodology, where one DDD for lithium corresponds to 24 mmol (approximately 900 mg) of lithium carbonate, the average maintenance dose per day for its main indication in adults [19–21]. In Spain, lithium is typically dispensed in 400 mg tablets, resulting in common daily doses of 800 mg. A patient taking 800 mg daily for a year would accumulate roughly 324 DDDs. Since DDDs in our dataset were recorded in increments of 50, we defined sustained use as ≥350 DDDs per year, allowing for minor interruptions while reflecting consistent maintenance therapy over ≥11.5 months annually. Patients with sustained lithium exposure consistently met this threshold from their first prescription until the end of follow-up (2019) or death. For individuals who died during follow-up, the final year was exempt from the 350-DDD requirement, provided they received some lithium in that year. Partial or intermittent lithium exposure referred to patients who received lithium at some point during the study period but did not consistently reach the annual threshold of 350 DDDs. This group included individuals with irregular treatment patterns, such as interruptions between years, early discontinuation, or annual prescriptions below the defined threshold, resulting in short-term, sporadic, or low cumulative exposure. Patients with no lithium exposure had no recorded lithium prescriptions during the study period.

This classification aligns with previous approaches, adapting WHO DDD standards to psychiatric indications while acknowledging variations in clinical practice [22]. Applying a consistent threshold helped distinguish distinct exposure trajectories and reduce misclassification due to brief or sporadic use.

The primary outcome was time to all-cause mortality, defined as the period from January 1, 2015, to death or censoring on December 31, 2019, based on linked electronic health records.

### Statistical analysis

Descriptive statistics were calculated to summarize baseline characteristics, using means and standard deviations (SDs) for continuous variables and frequencies and percentages for categorical variables. Baseline characteristics included age, sex, BD type, socioeconomic level, somatic comorbidities, and combination with other psychotropic medications (mood stabilizers, antidepressants, and antipsychotics).

To compare age across lithium exposure groups (sustained, partial or intermittent, and none), we performed one-way ANOVA, with eta squared (η²) as the effect size. Categorical variables were compared using chi-square tests, with Cramér’s V to quantify effect sizes. When associations were significant (*p* < 0.05), post-hoc pairwise comparisons were conducted: Tukey’s HSD for continuous variables and chi-square tests with Bonferroni correction for categorical ones.

Kaplan–Meier survival curves were generated to compare survival across exposure groups. The log-rank test was used to assess differences, with Bonferroni-adjusted pairwise comparisons.

Cox proportional hazards regression models estimated hazard ratios (HRs) and 95% confidence intervals (CIs) for the association between lithium exposure and all-cause mortality. Three models were applied: an unadjusted model including only lithium exposure; a minimally adjusted model controlling for age and sex; and a fully adjusted model accounting for sociodemographic factors (age, sex, socioeconomic level), clinical characteristics (BD type, and somatic comorbidities), and treatment variables (combination with other mood stabilizers, antidepressants, and antipsychotics). The proportional hazards assumption was verified using Schoenfeld residuals. A subgroup analysis compared sustained versus partial or intermittent lithium exposure. Sensitivity analyses tested alternative DDD thresholds (≥150, 200, 250, 300, and 400 per year) to assess the robustness of exposure classification. For each threshold, we re-ran Kaplan–Meier and fully adjusted Cox models.

All analyses were performed using R version 4.4.1.

## Results

The completed STROBE checklist is available in the Supplementary Appendix (p. 2).

The cohort included 15,384 individuals with a diagnosis of BD. Table 1 summarizes the distribution of the variables considered in the sample. During the study period, 8,815 (57.3%) patients were classified as lithium non-users, 4,331 (28.15%) as discontinuous lithium users, and 2,238 (14.55%) as continuous lithium users.

**Table 1.** Baseline characteristics of the cohort of patients with bipolar disorders (n = 15,384)

Variable		N/mean (%/SD)
Age		44.42 (21.6)
Sex	Males	6461 (42)
	Females	8923 (58)
Bipolar disorder type	BD-I	13073 (85)
	BD-II	2144 (13.9)
	BD-NOS	167 (1.1)
Socioeconomic level	< 18,000 €	11979 (77.9)
	18,001 – 100,000 €	3333 (21.7)
	> 100,000 €	72 (0.5)
Somatic comorbidities	0	8062 (52.4)
	1	3466 (22.5)
	≥ 2	3856 (25.1)
Combination with mood stabilizers	Yes	11477 (74.6)
	No	3907 (25.4)
Combination with antidepressants	Yes	4023 (26.2)
	No	11361 (73.8)
Combination with antipsychotics	Yes	3015 (19.6)
	No	12369 (80.4)

Differences across the three groups are reported in Table 2. The one-way ANOVA indicated that there was no statistically significant difference in age between the groups. Significant differences between groups were found for sex distribution, BD type, socioeconomic level, presence of somatic comorbidities, and concomitant medications use. Pairwise comparisons between the three groups are reported in Table 3.

**Table 2.** Baseline characteristics of patients with bipolar disorders stratified by lithium exposure patterns

		No lithium exposure, n = 8815, N/mean (%/SD)	Partial or intermittent lithium exposure, n = 4331, N/mean (%/SD)	Sustained lithium exposure, n = 2238, N/mean (%/SD)	F/Chi-square statistic	Degrees of freedom (df)	*p*-value	η²/Cramér’s V
Age		44.18 (21.77)	44.41 (21.49)	45.37 (21.27)	2.72	2	0.06	0.001
Sex	Males	3560 (40.4)	1804 (41.6)	1097 (49.2)	54.88	2	<0.001	0.06
	Females	5255 (59.6)	2527 (58.4)	1141 (50.9)				
Bipolar disorder type	BD-I	7542 (85.6)	3610 (83.4)	1921 (85.8)	41.49	4	<0.001	0.037
	BD-II	1209 (13.7)	650 (15)	285 (12.8)				
	BD-NOS	64 (0.7)	71 (1.6)	32 (1.4)				
Socioeconomic level	< 18,000 €	6913 (78.4)	3352 (77.4)	1714 (76.6)	23.4	4	<0.001	0.028
	18,001 – 100,000 €	1876 (21.3)	956 (22.1)	501 (22.4)				
	> 100,000 €	26 (0.3)	23 (0.5)	23 (1)				
Somatic comorbidities	0	4447 (50.4)	2197 (50.7)	1418 (63.4)	205.07	4	<0.001	0.082
	1	1975 (22.4)	969 (22.4)	522 (23.3)				
	≥ 2	2393 (27.1)	1165 (26.9)	298 (13.3)				
Combination with mood stabilizers	Yes	2204 (25)	1037 (23.9)	666 (29.7)	82.02	2	<0.001	0.043
	No	6611 (75)	3294 (76.1)	1572 (70.3)				
Combination with antidepressants	Yes	2069 (23.5)	1199 (27.7)	755 (33.7)	104.7	2	<0.001	0.082
	No	6746 (76.5)	3132 (72.3)	1572 (66.3)				
Combination with antipsychotics	Yes	2196 (24.9)	559 (12.9)	260 (11.6)	371.48	2	<0.001	0.155
	No	6619 (75.1)	3772 (87.1)	1978 (88.4)

**Table 3.** Pairwise comparisons for categorical variables (chi-square tests)

Variable (reference)	Comparison	Chi-square	Degrees of freedom (df)	Adjusted *p-*value	Cramér’s V
Sex (females)	No lithium exposure versus partial or intermittent lithium exposure	1.88	1	0.1704	0.012
	No lithium exposure versus sustained lithium exposure	54.18	1	<0.001	0.07
	Partial or intermittent versus sustained lithium exposure	32.15	1	<0.001	0.07
Bipolar disorder type (BD-I)	No lithium exposure versus partial or intermittent lithium exposure	35.65	2	<0.001	0.052
	No lithium exposure versus sustained lithium exposure	17.84	2	<0.001	0.04
	Partial or intermittent versus sustained lithium exposure	6.63	2	0.0364	0.032
Socioeconomic level (<18,000 €)	No lithium exposure versus partial or intermittent lithium exposure	5.58	2	0.0614	0.021
	No lithium exposure versus sustained lithium exposure	23.4	2	<0.001	0.046
	Partial or intermittent versus sustained lithium exposure	5.39	2	0.0676	0.029
Somatic comorbidities (0)	No lithium exposure versus partial or intermittent lithium exposure	0.11	2	0.9456	0.003
	No lithium exposure versus sustained lithium exposure	197	2	<0.001	0.134
	Partial or intermittent versus sustained lithium exposure	165.62	2	<0.001	0.159
Combination with Mood stabilizers (No)	No lithium exposure versus Partial or intermittent lithium exposure	1.7	1	0.1926	0.012
	No lithium exposure versus sustained lithium exposure	20.75	1	<0.001	0.044
	Partial or intermittent versus sustained lithium exposure	25.68	1	<0.001	0.063
Combination with antidepressants (No)	No lithium exposure versus partial or intermittent lithium exposure	27.37	1	<0.001	0.046
	No lithium exposure versus sustained lithium exposure	98.32	1	<0.001	0.095
	Partial or intermittent versus sustained lithium exposure	25.57	1	<0.001	0.063
Combination with antipsychotics (No)	No lithium exposure versus partial or intermittent lithium exposure	251.95	1	<0.001	0.139
	No lithium exposure versus sustained lithium exposure	181.76	1	<0.001	0.128
	Partial or intermittent versus sustained lithium exposure	2.13	1	0.1443	0.018

Over the study period, 715 deaths were recorded in our cohort of patients with BD. Among these, 467 (65.3%) deaths occurred in patients with no lithium exposure, 199 (27.8%) in those with partial or intermittent exposure, and 49 (6.9%) in those with sustained lithium exposure. The overall mortality rate was 10.16 per 1,000 person-years (95% CI: 9.44–10.93). Mortality rates were 10.9 per 1,000 person-years (95% CI: 9.91–11.9) in the no-exposure group, 10.5 (95% CI: 9.11–12.0) in the partial or intermittent exposure group, and substantially lower at 5.87 (95% CI: 4.43–7.76) in the sustained exposure group.

**Figure 1.**
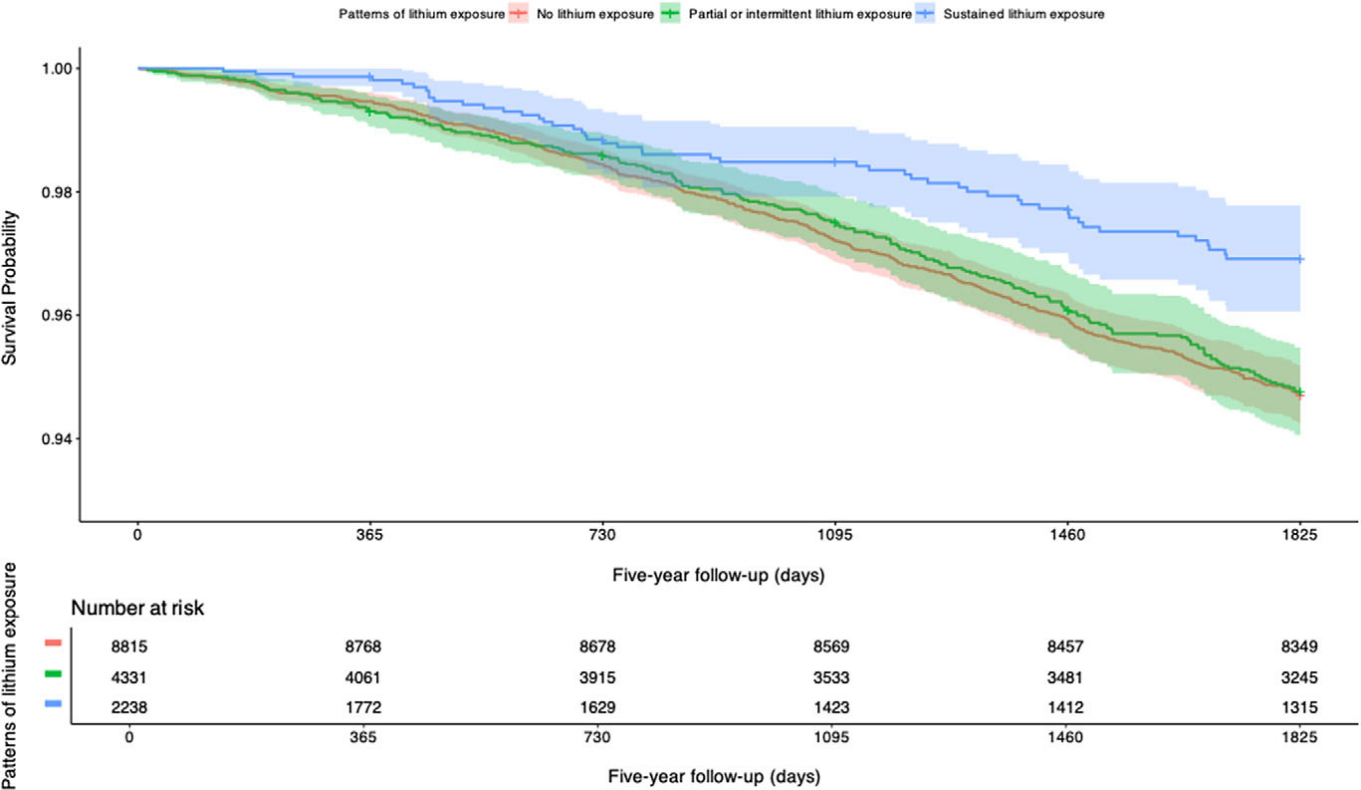
Kaplan-Meier survival curves stratified by lithium exposure. Kaplan–Meier survival curves illustrating the probability of survival over time, stratified by patterns of lithium exposure: no exposure, partial or intermittent exposure, and sustained exposure. Survival probabilities are shown from January 1, 2015, with censoring events indicated by marks on the curves. Shaded areas represent 95% confidence intervals.

Figure 1 presents Kaplan–Meier survival curves stratified by lithium use. The log-rank test demonstrated a significant difference among the three survival curves (χ^2^ = 14.8; *p* < 0.001). The pairwise log-rank test, adjusted for multiple comparisons using the Bonferroni method, revealed significant differences in survival curves. Specifically, patients with sustained lithium exposure showed significantly higher survival probabilities compared to those with no lithium exposure (*p* < 0.001) and those with partial or intermittent exposure (*p* = 0.001). No significant difference was observed between the partial or intermittent exposure group and the no-exposure group (*p* = 1).

Table 4 presents the results of the Cox proportional hazards models examining the association between lithium use patterns and all-cause mortality. In the unadjusted model, sustained lithium use was associated with a significantly lower risk of mortality compared to no lithium exposure. This association remained significant after adjusting for age and sex, and persisted in the fully adjusted model accounting for sociodemographic, clinical, and pharmacological covariates. In contrast, discontinuous lithium use was not significantly associated with mortality in any model. Age was not a significant predictor of mortality in the adjusted models. Female sex was consistently associated with a lower mortality risk across both the minimally and fully adjusted models. Among covariates, the presence of somatic comorbidities emerged as the strongest predictor of increased mortality: individuals with one comorbidity had a more than threefold increased risk, while those with two or more comorbidities had over a 10-fold increase in risk. Antipsychotic use was also independently associated with a higher risk of death. Mid-level socioeconomic status was associated with a significantly lower mortality risk compared to the lowest-income group, whereas no significant difference was observed for the highest-income category. Other covariates, including BD type, antidepressant use, and mood stabilizer use, were not significantly associated with mortality. The model’s concordance index was 0.77, indicating good discriminative ability. The proportional hazards assumption was met (global test: χ² = 9.52, df = 13, *p* = 0.73). Full results of the Schoenfeld residual tests are available in the Supplementary Appendix (p. 4).

**Table 4.** Cox proportional hazards models for all-cause mortality according to lithium use patterns

Variable		Unadjusted model HR (95%CI), p-value	Minimally adjusted model HR (95%CI), p-value	Fully adjusted model HR (95%CI), p-value
Partial or intermittent lithium exposure versus no lithium exposure		0.98 (0.83–1.16), 0.85	0.98 (0.83–1.16), 0.85	0.91 (0.77–1.08), 0.28
Sustained lithium exposure versus no lithium exposure		**0.57 (0.42–0.76), <0.001**	**0.54 (0.4–0.73), <0.001**	**0.69 (0.51–0.93), 0.02**
Age		–	1.00 (0.99–1.01), 0.52	1.00 (0.996–1.003), 0.82
Sex (females versus males)		–	**0.66 (0.57–0.76), <0.001**	**0.67 (0.58–0.77), <0.001**
Socioeconomic status	(18,001 – 100,000€ versus < 18,000€)	–	–	**0.73 (0.59–0.89), 0.002**
	(> 100,000€ versus < 18,000€)			1.65 (0.61–4.42), 0.32
BD type	(BD-II versus BD-I)	–	–	0.85 (0.68–1.07), 0.17
	(BD-NOS Vs BD-I)			0.48 (0.15–1.5), 0.2
Comorbidities	(1 Vs 0)	–	–	**3.36 (2.58–4.39), <0.001**
	(≥2 versus 0)			**10.72 (8.56–13.43), <0.001**
Combination with antipsychotics (yes versus no)		–	–	**1.64 (1.29–2.07), <0.001**
Combination with antidepressants (yes versus no)		–	–	0.88 (0.73–1.06), 0.17
Combination with mood stabilizers (yes versus no)		–	–	0.94 (0.78–1.14), 0.52

*Note:* Significant results are reported in bold.

In the subgroup analysis restricted to patients with lithium exposure, those with sustained lithium exposure had a significantly lower risk of all-cause mortality compared to those with partial or intermittent exposure (HR = 0.70, 95% CI: 0.51–0.97, p = 0.03). Significant predictors of increased mortality included the presence of comorbidities, with a twofold increase in risk for patients with one comorbidity (HR = 2.46, 95% CI: 1.66–3.63, p < 0.001), and over a sixfold increase for those with two or more comorbidities (HR = 6.50, 95% CI: 4.67–9.04, p < 0.001). Female sex remained independently associated with a lower mortality risk (HR = 0.57, 95% CI: 0.44–0.73, p < 0.001), and individuals with mid-level socioeconomic status had a reduced risk compared to those in the lowest-income group (HR = 0.66, 95% CI: 0.47–0.94, p = 0.02). Other variables, including age (HR = 1.00, 95% CI: 0.99–1.00, p = 0.42), BD type (BD-II: HR = 0.67, 95% CI: 0.45–1.02, p = 0.06; BD-NOS: HR = 0.31, 95% CI: 0.04–2.24, p = 0.25), and the use of antidepressants (HR = 0.89, 95% CI: 0.67–1.18, p = 0.42), antipsychotics (HR = 1.42, 95% CI: 0.91–2.20, p = 0.12), or mood stabilizers (HR = 1.08, 95% CI: 0.78–1.48, p = 0.65), were not significantly associated with mortality.

The results of the sensitivity analyses across different DDD thresholds are presented in Table 5, reporting HRs and 95% CIs from fully adjusted Cox regression models. The protective association of sustained lithium exposure with reduced all-cause mortality was consistently confirmed at lower DDD thresholds, but this association lost significance as the DDD threshold increased. Corresponding Kaplan–Meier survival curves and regression outputs for each threshold are available in the Supplementary Appendix (pp. 5–6).

**Table 5.** Sensitivity analysis results using different thresholds of defined daily doses (DDDs) for categorizing lithium exposure patterns

Defined daily dose threshold	Exposure	(n, %)	Log-rank test (p-value)	Cox regressions
≥150	No lithium exposure	8815(57.3%)	81 (<0.001)	Ref
	Partial or intermittent lithium exposure	2577 (16.7%)		**1.28 (1.07‚ 1.52)**
	Sustained lithium exposure	3992 (26%)		**0.47 (0.37‚ 0.6)**
≥200	No lithium exposure	8815 (57.3%)	56.9 (<0.001)	Ref
	Partial or intermittent lithium exposure	2926 (19%)		1.13 (0.95‚ 1.35)
	Sustained lithium exposure	3643 (23.7%)		**0.52 (0.4‚ 0.3)**
≥250	No lithium exposure	8815 (57.3%)	52.6 (<0.001)	Ref
	Partial or intermittent lithium exposure	3319 (21.6%)		1.08 (0.91‚ 1.28)
	Sustained lithium exposure	3250 (21.1%)		**0.49 (0.38 0.65)**
≥300	No lithium exposure	8815 (57.3%)	28.8 (<0.001)	Ref
	Partial or intermittent lithium exposure	3791 (24.6%)		0.99 (0.83‚ 1.17)
	Sustained lithium exposure	2778 (18.1%)		**0.58 (0.44‚ 0.77)**
≥400	No lithium exposure	8815 (57.3%)	7.5 (0.02)	Ref
	Partial or intermittent lithium exposure	4917 (32%)		0.86 (0.73‚ 1.02)
	Sustained lithium exposure	1652 (10.7%)		0.84 (0.6‚ 1.18)

*Note:* This table presents the sensitivity analysis results for alternative DDD thresholds used to classify lithium use patterns. Cox proportional hazards regression models were used to estimate hazard ratios (HRs) and 95% confidence intervals (CIs) for mortality risk, with lithium non-users as the reference category. Significant results are highlighted in bold.

Given the strong association between medical comorbidities and all-cause mortality observed in our primary analysis, and the potential for confounding by indication, we conducted a post-hoc Cox regression model including interaction terms between patterns of lithium exposure and comorbidity burden (none, one, or two or more comorbidities). This analysis aimed to explore whether the observed protective effect of sustained lithium exposure might reflect an underlying health selection effect – that is, whether healthier individuals are more likely to maintain long-term lithium treatment. The interaction between sustained lithium exposure and comorbidity status was not statistically significant. Among patients with one comorbidity, sustained lithium exposure was associated with a non-significant reduction in mortality risk (HR = 0.57, 95% CI: 0.24–1.34, p = 0.19) and similarly among those with two or more comorbidities (HR = 0.49, 95% CI: 0.23–1.03, p = 0.06). In contrast, a significant interaction was found for partial or intermittent lithium exposure. Among patients with no comorbidities, partial or intermittent lithium exposure was associated with a significantly increased risk of all-cause mortality compared to no lithium exposure (HR = 1.86, 95% CI: 1.20–2.90, p = 0.006). This elevated risk appeared to diminish in patients with one comorbidity (interaction HR = 0.55, 95% CI: 0.31–0.98, p = 0.04) and was further reduced in those with two or more comorbidities (interaction HR = 0.41, 95% CI: 0.25–0.67, p < 0.001). Full model results, including all interaction terms and stratified HRs, are reported in the Supplementary Appendix (p. 7).

## Discussion

This study provides novel evidence that the pattern of lithium exposure – whether sustained or partial/intermittent – significantly affects mortality in individuals with BD. Using a large population-based cohort, we found that sustained lithium exposure was associated with a significantly lower risk of all-cause mortality compared to both no exposure and partial or intermittent exposure. Specifically, sustained users had a 31% lower mortality risk than non-users and a 30% lower risk than those with partial or intermittent exposure. To our knowledge, this is the first study to systematically evaluate the impact of lithium treatment continuity on all-cause mortality in a real-world population of individuals with BD. A post-hoc interaction analysis showed no significant interaction between sustained lithium exposure and comorbidity burden, suggesting that this protective effect is not merely due to healthier individuals being more likely to remain on treatment.

These results align with prior studies showing reduced mortality with lithium compared to placebo or other treatments [6, 8, 13]. Several mechanisms may explain this beneficial effect. Clinical research has demonstrated that lithium treatment is associated with a reduced risk of cardiovascular disease in individuals with BD [23]. Additionally, preclinical studies using cellular and animal models of myocardial infarction suggest that lithium, when administered at therapeutic levels, may mitigate pathological hypertrophy and cardiac fibrosis [24]. Its cardioprotective effects are thought to be mediated through the modulation of inflammatory pathways, oxidative stress reduction, and the preservation of endothelial function, involving various molecular signaling mechanisms [25]. Furthermore, beneficial effects on neuronal plasticity and neuroinflammatory modulation may contribute to improved overall health and longevity [26]. These biological effects of lithium, combined with its well-established efficacy in preventing relapse of any mood episode [27], treating manic episodes [28], and its anti-suicidal properties [5], continue to make it a first-line treatment in the management of BD [4].

Sensitivity analyses confirmed the robustness of our findings across varying DDD thresholds. Lowering the threshold expanded the group of sustained users and restricted the partial/intermittent group to those with truly irregular exposure. While the protective effect on mortality remained consistent for sustained lithium users, individuals with partial or intermittent exposure exhibited an increased mortality risk when the lowest DDD threshold was applied. These findings suggest that treatment continuity may play a critical role in maximizing lithium’s protective effect on survival. This is consistent with prior research showing that discontinuing lithium is associated with a higher mortality risk than discontinuing valproate [13], supporting the notion that lithium discontinuation may have more detrimental effects on survival than non-use. While the precise mechanisms underlying this association remain unclear, they may extend beyond lithium’s established anti-suicidal properties to broader benefits for physical health when used consistently over time. Conversely, at higher thresholds (≥400 DDD/year), the protective effect of sustained exposure diminished, possibly due to dose-dependent toxicity offsetting benefits. Higher lithium doses are associated with increased risk of renal and thyroid dysfunction, cardiovascular burden, and other adverse effects, particularly in older or medically vulnerable patients. These risks may attenuate the survival benefits observed at moderate doses, suggesting the existence of a therapeutic window in which lithium confers maximal protection. This finding highlights the importance of individualized dosing and regular monitoring to balance efficacy and tolerability in long-term treatment strategies [26].

Our post-hoc analyses also revealed that the excess mortality associated with partial or intermittent lithium exposure was particularly marked among individuals without comorbidities. This suggests that abrupt or inconsistent use may be especially detrimental in otherwise physically healthy individuals, while the relative impact is less visible in those with high baseline mortality risk due to medical burden.

Taken together, these findings underscore the clinical importance of maintaining lithium continuity. Once initiated, lithium treatment should be actively monitored and, when clinically appropriate, maintained over time. Ensuring long-term adherence is key to maximize both psychiatric and physical health outcomes [29].

Beyond lithium use patterns, our Cox regression models identified additional factors influencing mortality risk. The presence of somatic comorbidities was the strongest predictor of increased mortality, with an HR of 3.36 for one comorbidity and 10.32 for two or more comorbidities. These findings emphasize the substantial impact of medical burden on survival in BD [30], reinforcing the need for integrated medical and psychiatric care [31]. Antipsychotic use was also associated with increased mortality, consistent with prior evidence linking these medications to physical health risks [32]. While essential for managing acute symptoms, antipsychotics require close monitoring, particularly in high-risk patients [4]. Consistent with prior studies, female sex was associated with a lower risk of mortality [33], likely reflecting lower suicide rates [34], better healthcare-seeking behaviors [35], and biological differences in disease progression [36, 37]. Furthermore, patients with mid-level socioeconomic status had a lower mortality risk compared to those in the lowest socioeconomic group, suggesting that socioeconomic factors play a crucial role in treatment access, adherence, and overall health outcomes [38].

Although significant differences in baseline characteristics were observed across lithium groups, effect sizes were generally small (Cramér’s V < 0.2), consistent with large sample studies where minor differences often reach statistical significance [39, 40]. The most notable group differences involved antipsychotic use and comorbidities, though effect sizes were weak. For antipsychotic use, the overall Cramér’s V was 0.155, with pairwise values of 0.139 (no exposure versus partial/intermittent) and 0.128 (no exposure versus sustained); no differences emerged between lithium-exposed groups. For comorbidities, weak associations were found between no exposure and sustained exposure (Cramér’s V = 0.134) and between partial/intermittent and sustained exposure (Cramér’s V = 0.159).

A major strength of this study is the use of a large, population-based cohort with longitudinal prescription and mortality data, enabling a robust evaluation of lithium exposure patterns and their association with all-cause mortality over a 10-year period. By leveraging real-world data from all levels of psychiatric care within a universal healthcare system, the study enhances the external validity of its findings and supports their generalisability to routine clinical practice. The detailed categorization of lithium exposure, combined with rigorous sensitivity analyses, further strengthens the reliability and interpretability of our results. Finally, the interaction analysis with comorbidity burden provides novel insight into how the association between lithium use and mortality may vary across different clinical profiles, thereby reducing residual confounding and supporting a more nuanced causal interpretation. However, certain limitations should be acknowledged. First, lithium exposure was inferred from prescription data, which does not account for actual medication intake, adherence, or potential dose adjustments [41]. While DDD provide a standardized measure of drug exposure, they may not fully capture individual variations in prescribing practices, such as dose titration, temporary discontinuations, or therapeutic drug monitoring adjustments. Indeed, DDDs reflect the amount of drug dispensed, not necessarily consumed, and do not account for whether patients actually took the medication as prescribed. Therefore, they approximate adherence but cannot measure it directly. However, they offer a useful proxy for long-term treatment continuity when aggregated over time. Using prescription records offers an objective and reproducible method to assess long-term lithium exposure in a large, real-world population, reducing recall bias and providing valuable insights into treatment patterns and outcomes. This approach has been widely used in previous pharmacoepidemiologic studies on psychopharmacology [22]. Second, unmeasured confounders may influence both lithium adherence and mortality risk. While our study adjusted for possible key demographic and clinical covariates, residual confounding remains possible. Third, this study should be considered a post-hoc analysis, as the data were collected without a predefined hypothesis [42]. Consequently, its conclusions should be interpreted as preliminary and require confirmation in longitudinal studies specifically designed to evaluate the association between lithium use patterns and mortality [43]. Fourth, potential heterogeneity within the study population, including differences in illness severity, treatment history, and comorbidities, may have influenced the observed associations despite statistical adjustments [44]. Fifth, specific causes of death were not available in our dataset. This precluded analyses of whether lithium’s protective effects vary by cause of death – such as cardiovascular, metabolic, or suicide-related mortality – which would be important to clarify the underlying mechanisms. Finally, as the study was conducted within a single public healthcare system in Catalonia, Spain, replication in other settings is needed to confirm the external validity and generalizability of our findings.

Further research should aim to better characterize individuals with partial or intermittent lithium exposure and identify strategies to improve treatment continuity. Prospective studies incorporating biomarkers, genetic profiles, and real-time adherence monitoring could help clarify the mechanisms behind the observed mortality differences. Future work should also explore the relationship between lithium exposure patterns and specific causes of death, especially cardiovascular and metabolic conditions.

## Conclusions

This study reinforces the critical role of sustained lithium treatment in improving survival in individuals with BD. Sustained lithium exposure was consistently associated with reduced all-cause mortality, whereas partial or intermittent exposure conferred no benefit and may entail additional risks. These findings support clinical strategies that prioritize treatment continuity and long-term adherence to optimize both psychiatric and physical health outcomes in BD.

## Supporting information

10.1192/j.eurpsy.2025.10090.sm001 Oliva et al. supplementary material Oliva et al. supplementary material

## Data Availability

The dataset used and analyzed in this study will be available upon reasonable request from the corresponding author, in accordance with the agreement between PADRIS-AQuAS and Hospital Clínic of Barcelona.

## Supplementary material

The supplementary material for this article can be found at http://doi.org/10.1192/j.eurpsy.2025.10090.

## References

[1] Merikangas KR, Jin R, He JP, Kessler RC, Lee S, Sampson NA, et al. Prevalence and correlates of bipolar spectrum disorder in the world mental health survey initiative. Arch Gen Psychiatry. 2011;68(3):241–51. 10.1001/archgenpsychiatry.2011.12. 21383262 PMC3486639

[2] Oliva V, Fico G, De Prisco M, Gonda X, Rosa AR, Vieta E. Bipolar disorders: An update on critical aspects. Lancet Reg Health Eur. 2025;48:101135. 39811787 10.1016/j.lanepe.2024.101135 PMC11732062

[3] Kessing LV, Ziersen SC, Andersen PK, Vinberg M. A nation-wide population-based longitudinal study on life expectancy and cause specific mortality in patients with bipolar disorder and their siblings. J Affect Disord. 2021;294:472–6. 34325167 10.1016/j.jad.2021.07.065

[4] Yatham LN, Kennedy SH, Parikh SV, Schaffer A, Bond DJ, Frey BN, et al. Canadian network for mood and anxiety treatments (CANMAT) and International Society for Bipolar Disorders (ISBD) 2018 guidelines for the management of patients with bipolar disorder. Bipolar Disord. 2018;20(2):97–170. 29536616 10.1111/bdi.12609 PMC5947163

[5] Smith KA, Cipriani A. Lithium and suicide in mood disorders: Updated meta‐review of the scientific literature. Bipolar Disord. 2017;19(7):575–86. 28895269 10.1111/bdi.12543

[6] Cipriani A, Pretty H, Hawton K, Geddes JR. Lithium in the prevention of suicidal behavior and all-cause mortality in patients with mood disorders: A systematic review of randomized trials. Am J Psychiatry. 2005;162(10):1805–19. 16199826 10.1176/appi.ajp.162.10.1805

[7] Del Matto L, Muscas M, Murru A, Verdolini N, Anmella G, Fico G, et al. Lithium and suicide prevention in mood disorders and in the general population: A systematic review. Neurosci Biobehav Rev. 2020;116:142–53. 10.1016/j.neubiorev.2020.06.017. 32561344

[8] Chen PH, Tsai SY, Chen PY, Pan CH, Su SS, Chen CC, et al. Mood stabilizers and risk of all‐cause, natural, and suicide mortality in bipolar disorder: A nationwide cohort study. Acta Psychiatr Scand. 2023;147(3):234–47. 36367926 10.1111/acps.13519

[9] Carvalho AF, Hsu CW, Vieta E, Solmi M, Marx W, Berk M, et al. Mortality and lithium-protective effects after first-episode mania diagnosis in bipolar disorder: A Nationwide retrospective cohort study in Taiwan. Psychother Psychosom. 2024;93(1):36–45. 10.1159/000535777. 38194936 PMC10880805

[10] Toffol E, Hätönen T, Tanskanen A, Lönnqvist J, Wahlbeck K, Joffe G, et al. Lithium is associated with decrease in all-cause and suicide mortality in high-risk bipolar patients: A nationwide registry-based prospective cohort study. J Affect Disord. 2015;183:159–65. 26005778 10.1016/j.jad.2015.04.055

[11] Baldessarini RJ, Pinna M, Contu M, Vázquez GH, Tondo L. Risk factors for early recurrence after discontinuing lithium in bipolar disorder. Bipolar Disord. 2022;24(7):720–5. 10.1111/bdi.13206. 35319801

[12] Tondo L, Baldessarini RJ. Prevention of suicidal behavior with lithium treatment in patients with recurrent mood disorders. Int J Bipolar Disord. 2024;12(1):6 10.1186/s40345-024-00326-x. 38460088 PMC10924823

[13] Smith EG, Austin KL, Kim HM, Eisen SV, Kilbourne AM, Miller DR, et al. Mortality associated with lithium and valproate treatment of US veterans health administration patients with mental disorders. Br J Psychiatry. 2015;207(1):55–63. 10.1192/bjp.bp.113.138685. 25953891

[14] AQuAS. Programa d’analítica de dades per a la recerca i la innovació en salut (PADRIS), https://aquas.gencat.cat/ca/fem/intelligencia-analitica/padris/; 2020 [accessed 8 December 2024].

[15] Mas A, Clougher D, Anmella G, Valenzuela-Pascual C, De Prisco M, Oliva V, et al. Trends and associated factors of mental health diagnoses in Catalan primary care (2010–2019). Eur Psychiatry. 2024;67(1):e81 10.1192/j.eurpsy.2024.1793. 39655694 PMC11733616

[16] Anmella G, Sanabra M, Primé-Tous M, Segú X, Solanes A, Ruíz V, et al. Antidepressants overuse in primary care: Prescription trends between 2010 and 2019 in Catalonia. Rev Psiquiatr Salud Ment. 2022. 10.1016/j.rpsm.2022.12.001. 37758595

[17] Anmella G, Primé-Tous M, Segú X, Solanes A, Ruíz V, Martín-Villalba I, et al. PRimary carE digital support ToOl in mental health (PRESTO): Design, development and study protocols. Span J Psychiatry Ment Health. 2024;17(2):114–25. 33933665 10.1016/j.rpsm.2021.04.003

[18] Cuschieri S. The STROBE guidelines. Saudi J Anaesth. 2019;13(Suppl 1):S31–S4. 30930717 10.4103/sja.SJA_543_18 PMC6398292

[19] Grandjean EM, Aubry J-M. Lithium: Updated human knowledge using an evidence-based approach: Part II: Clinical pharmacology and therapeutic monitoring. CNS Drugs. 2009;23:331–49. 19374461 10.2165/00023210-200923040-00005

[20] WHO. WHO Collaborating Centre for Drug Statistics Methodology. ATC/DDD Index 2024: N05AN01 Lithium, https://atcddd.fhi.no/atc_ddd_index/?code=N05AN01; 2024 [accessed 21 March 2025].

[21] Ronning M. A historical overview of the ATC/DDD methodology. WHO Drug Inf. 2002;16(3):233.

[22] Kendler KS, Ohlsson H, Sundquist J, Sundquist K. The relationship between familial-genetic risk and pharmacological treatment in a Swedish national sample of patients with major depression, bipolar disorder, and schizophrenia. Mol Psychiatry. 2024;29(3):742–9. 38123723 10.1038/s41380-023-02365-9

[23] Tsai S-Y, Shen R-S, Kuo C-J, Chen P-H, Chung K-H, Hsiao C-Y, et al. The association between carotid atherosclerosis and treatment with lithium and antipsychotics in patients with bipolar disorder. Aust N Z J Psychiatry. 2020;54(11):1125–34. 32900219 10.1177/0004867420952551

[24] Lee T-M, Lin S-Z, Chang N-C. Effect of lithium on ventricular remodelling in infarcted rats via the Akt/mTOR signalling pathways. Biosci Rep. 2017;37(2):BSR20160257. 28115595 10.1042/BSR20160257 PMC5469250

[25] Kim S, Bong N, OS , Jin J, Kim DE, Lee DK. Lithium chloride suppresses LPS‐mediated matrix metalloproteinase‐9 expression in macrophages through phosphorylation of GSK‐3β. Cell Biol Int. 2015;39(2):177–84. 25053111 10.1002/cbin.10352

[26] Bortolozzi A, Fico G, Berk M, Solmi M, Fornaro M, Quevedo J, et al. New advances in the pharmacology and toxicology of lithium: A neurobiologically oriented overview. Pharmacol Rev. 2024;76(3):323–57. 38697859 10.1124/pharmrev.120.000007 PMC11068842

[27] Kishi T, Ikuta T, Matsuda Y, Sakuma K, Okuya M, Mishima K, et al. Mood stabilizers and/or antipsychotics for bipolar disorder in the maintenance phase: A systematic review and network meta-analysis of randomized controlled trials. Mol Psychiatry. 2021;26(8):4146–57. 33177610 10.1038/s41380-020-00946-6 PMC8550938

[28] Kishi T, Ikuta T, Matsuda Y, Sakuma K, Okuya M, Nomura I, et al. Pharmacological treatment for bipolar mania: A systematic review and network meta-analysis of double-blind randomized controlled trials. Mol Psychiatry. 2022;27(2):1136–44. 10.1038/s41380-021-01334-4. 34642461 PMC9054678

[29] Berk L, Hallam KT, Colom F, Vieta E, Hasty M, Macneil C, et al. Enhancing medication adherence in patients with bipolar disorder. Hum Psychopharmacol Clin Exp. 2010;25(1):1–16. 10.1002/hup.1081 20041478

[30] Biazus TB, Beraldi GH, Tokeshi L, LdS R, Dragioti E, Carvalho AF, et al. All-cause and cause-specific mortality among people with bipolar disorder: A large-scale systematic review and meta-analysis. Mol Psychiatry. 2023;28(6):2508–24. 37491460 10.1038/s41380-023-02109-9 PMC10611575

[31] Liu NH, Daumit GL, Dua T, Aquila R, Charlson F, Cuijpers P, et al. Excess mortality in persons with severe mental disorders: A multilevel intervention framework and priorities for clinical practice, policy and research agendas. World Psychiatry. 2017;16(1):30–40. 10.1002/wps.20384. 28127922 PMC5269481

[32] Correll CU, Detraux J, De Lepeleire J, De Hert M. Effects of antipsychotics, antidepressants and mood stabilizers on risk for physical diseases in people with schizophrenia, depression and bipolar disorder. World Psychiatry. 2015;14(2):119–36. 10.1002/wps.20204. 26043321 PMC4471960

[33] Schumacher AE, Kyu HH, Aali A, Abbafati C, Abbas J, Abbasgholizadeh R, et al. Global age-sex-specific mortality, life expectancy, and population estimates in 204 countries and territories and 811 subnational locations, 1950–2021, and the impact of the COVID-19 pandemic: A comprehensive demographic analysis for the global burden of disease study 2021. Lancet. 2024;403(10440):1989–2056. 38484753 10.1016/S0140-6736(24)00476-8 PMC11126395

[34] Feigin VL, Vos T, Nair BS, Hay SI, Abate YH, Abd Al Magied AH, et al. Global, regional, and national burden of epilepsy, 1990–2021: A systematic analysis for the global burden of disease study 2021. Lancet Public Health. 2025;10(3):e203–e227. 40015291 10.1016/S2468-2667(24)00302-5 PMC11876103

[35] Ballering AV, Olde Hartman TC, Verheij R, Rosmalen JG. Sex and gender differences in primary care help-seeking for common somatic symptoms: A longitudinal study. Scand J Prim Health Care. 2023;41(2):132–9. 36995265 10.1080/02813432.2023.2191653 PMC10193899

[36] Serra-Navarro M, Clougher D, Oliva V, Valenzuela-Pascual C, De Prisco M, Forte MF, et al. Sex differences in psychosocial functioning and Neurocognition in bipolar disorder: A systematic review and meta-analysis. Eur Psychiatry. 2025;1–41. 10.1192/j.eurpsy.2025.27. PMC12041736 40040587

[37] Menculini G, Steardo Jr L, Sciarma T, D’Angelo M, Lanza L, Cinesi G, et al. Sex differences in bipolar disorders: Impact on psychopathological features and treatment response. Front Psych 2022;13:926594. 10.3389/fpsyt.2022.926594 PMC9226371 35757228

[38] Stringhini S, Carmeli C, Jokela M, Avendaño M, Muennig P, Guida F, et al. Socioeconomic status and the 25× 25 risk factors as determinants of premature mortality: A multicohort study and meta-analysis of 1· 7 million men and women. Lancet. 2017;389(10075):1229–37. 28159391 10.1016/S0140-6736(16)32380-7 PMC5368415

[39] Lee DK. Alternatives to P value: Confidence interval and effect size. Korean J Anesthesiol. 2016;69(6):555. 27924194 10.4097/kjae.2016.69.6.555 PMC5133225

[40] Sullivan GM, Feinn R. Using effect size—Or why the P value is not enough. J Grad Med Educ. 2012;4(3):279–82. 23997866 10.4300/JGME-D-12-00156.1 PMC3444174

[41] De Prisco M, Oliva V. As in cooking, so in medicine: Doses do matter. Eur Neuropsychopharmacol. 2023;69:24–5. 10.1016/j.euroneuro.2022.10.013. 36645957

[42] Oliva V, Vieta E. Predicting the past: The risks and rewards of post-hoc findings. Eur Neuropsychopharmacol. 2024;92:21–2. 10.1016/j.euroneuro.2024.12.005. 39709733

[43] Scott J, Hidalgo-Mazzei D, Strawbridge R, Young A, Resche-Rigon M, Etain B, et al. Prospective cohort study of early biosignatures of response to lithium in bipolar-I-disorders: Overview of the H2020-funded R-LiNK initiative. Int J Bipolar Disord. 2019;7(1):20 10.1186/s40345-019-0156-x. 31552554 PMC6760458

[44] Oliva V, De Prisco M. Together is better: Let’s overcome the heterogeneity problem. Eur Neuropsychopharmacol. 2022;65:33–4. 10.1016/j.euroneuro.2022.10.007. 36335785

