# Supplementary material for: Patterns of lithium exposure and mortality in bipolar disorder: A population-based cohort study
Source: Eur Psychiatry. 2025 Aug 27;68(1):e129. doi: 10.1192/j.eurpsy.2025.10090 (PMC12538171; doi:10.1192/j.eurpsy.2025.10090)
Supplement: Oliva et al. supplementary material [file S0924933825100904sup001.docx]

Summary

[Appendix 1. STROBE checklist 2](#_Toc198854102)

[Appendix 2. Schoenfeld residual test for the proportional hazards assumption. 4](#_Toc198854103)

[Appendix 3. Sensitivity Analysis, Kaplan-Meier Survival Curves at Different Defined Daily Dose (DDD) Thresholds 5](#_Toc198854104)

[Appendix 4. Cox proportional hazards regression results across different lithium use definitions based on defined daily dose (DDD) thresholds 6](#_Toc198854105)

[Appendix 5. Results from the Cox proportional hazards model assessing the interaction between lithium use patterns and medical comorbidities. 7](#_Toc198854106)

# Appendix 1. STROBE checklist

|  | **Item No** | **Recommendation** | **Pages** |
| --- | --- | --- | --- |
| **Title and abstract** | 1 | (*a*) Indicate the study’s design with a commonly used term in the title or the abstract | 1 |
|  |  | (*b*) Provide in the abstract an informative and balanced summary of what was done and what was found | 3 |
| **Introduction** | | |  |
| Background/rationale | 2 | Explain the scientific background and rationale for the investigation being reported | 4 |
| Objectives | 3 | State specific objectives, including any prespecified hypotheses | 4-5 |
| **Methods** | | |  |
| Study design | 4 | Present key elements of study design early in the paper | 5 |
| Setting | 5 | Describe the setting, locations, and relevant dates, including periods of recruitment, exposure, follow-up, and data collection | 5-6 |
| Participants | 6 | (*a*) Give the eligibility criteria, and the sources and methods of selection of participants. Describe methods of follow-up | 5-6 |
|  |  | (*b*) For matched studies, give matching criteria and number of exposed and unexposed | 5; 8 |
| Variables | 7 | Clearly define all outcomes, exposures, predictors, potential confounders, and effect modifiers. Give diagnostic criteria, if applicable | 5-7 |
| Data sources/ measurement | 8* | For each variable of interest, give sources of data and details of methods of assessment (measurement). Describe comparability of assessment methods if there is more than one group | 5 |
| Bias | 9 | Describe any efforts to address potential sources of bias | 7; 10-11 |
| Study size | 10 | Explain how the study size was arrived at | 5 |
| Quantitative variables | 11 | Explain how quantitative variables were handled in the analyses. If applicable, describe which groupings were chosen and why | 7 |
| Statistical methods | 12 | (*a*) Describe all statistical methods, including those used to control for confounding | 7-8 |
|  |  | (*b*) Describe any methods used to examine subgroups and interactions | 7-8 |
|  |  | (*c*) Explain how missing data were addressed | NA |
|  |  | (*d*) If applicable, explain how loss to follow-up was addressed | NA |
|  |  | (*e*) Describe any sensitivity analyses | 7-8 |
| **Results** | | |  |
| Participants | 13* | (a) Report numbers of individuals at each stage of study—eg numbers potentially eligible, examined for eligibility, confirmed eligible, included in the study, completing follow-up, and analysed | 8 |
|  |  | (b) Give reasons for non-participation at each stage | NA |
|  |  | (c) Consider use of a flow diagram | NA |
| Descriptive data | 14* | (a) Give characteristics of study participants (eg demographic, clinical, social) and information on exposures and potential confounders | Table1 |
|  |  | (b) Indicate number of participants with missing data for each variable of interest | NA |
|  |  | (c) Summarise follow-up time (eg, average and total amount) | 7 |
| Outcome data | 15* | Report numbers of outcome events or summary measures over time | Figure1 |
| Main results | 16 | (*a*) Give unadjusted estimates and, if applicable, confounder-adjusted estimates and their precision (eg, 95% confidence interval). Make clear which confounders were adjusted for and why they were included | 9-10 |
|  |  | (*b*) Report category boundaries when continuous variables were categorized | NA |
|  |  | (*c*) If relevant, consider translating estimates of relative risk into absolute risk for a meaningful time period | NA |
| Other analyses | 17 | Report other analyses done—eg analyses of subgroups and interactions, and sensitivity analyses | 10-11 |
| **Discussion** | | |  |
| Key results | 18 | Summarise key results with reference to study objectives | 11 |
| Limitations | 19 | Discuss limitations of the study, taking into account sources of potential bias or imprecision. Discuss both direction and magnitude of any potential bias | 14 |
| Interpretation | 20 | Give a cautious overall interpretation of results considering objectives, limitations, multiplicity of analyses, results from similar studies, and other relevant evidence | 11-13 |
| Generalisability | 21 | Discuss the generalisability (external validity) of the study results | 14 |
| **Other information** | | |  |
| Funding | 22 | Give the source of funding and the role of the funders for the present study and, if applicable, for the original study on which the present article is based | 3 |

# Appendix 2. Schoenfeld residual test for the proportional hazards assumption.

| **Variable** | **Chi-square** | **df** | **p-value** |
| --- | --- | --- | --- |
| Lithium exposure patterns | 0.35 | 2 | 0.84 |
| Mean age | 0.0238 | 1 | 0.88 |
| Sex | 0.5879 | 1 | 0.44 |
| Socioeconomic level | 1.2619 | 2 | 0.53 |
| Bipolar disorder type | 4.0944 | 2 | 0.13 |
| Combination with antidepressants | 0.5471 | 1 | 0.46 |
| Combination with antipsychotics | 0.4921 | 1 | 0.48 |
| Combination with mood stabilizers | 0.1256 | 1 | 0.72 |
| Somatic comorbidities | 2.4608 | 2 | 0.29 |
| GLOBAL | 9.5244 | 13 | 0.73 |

Chi-square statistics, degrees of freedom (df), and p-values are reported for each covariate in the fully adjusted Cox proportional hazards model. A non-significant p-value indicates no violation of the proportional hazards assumption. The global test result confirms the overall validity of the model (χ² = 9.52, df = 13, p = 0.73).

# Appendix 3. Sensitivity Analysis, Kaplan-Meier Survival Curves at Different Defined Daily Dose (DDD) Thresholds

**Legend:** The Kaplan-Meier survival curves illustrate the probability of survival over time stratified by lithium use categories: never, discontinuous, and continuous use. Survival probabilities are shown for the period from January 1, 2015, with events indicated by marks on the curves. Different cut-offs (**A** = DDD ≥ 150, **B** = DDD ≥ 200, **C** = DDD ≥ 250, **D** = DDD ≥ 300, **E** = DDD ≥ 400) are tested to assess the robustness of the findings. Shaded areas represent the 95% confidence intervals.

# Appendix 4. Cox proportional hazards regression results across different lithium use definitions based on defined daily dose (DDD) thresholds

| **Variable** | **≥ 150** | | **≥ 200** | | **≥ 250** | | **≥ 300** | | **≥ 400** | |
| --- | --- | --- | --- | --- | --- | --- | --- | --- | --- | --- |
|  | **HR** | **95% CI** | **HR** | **95% CI** | **HR** | **95% CI** | **HR** | **95% CI** | **HR** | **95% CI** |
| Partial or intermittent lithium exposure Vs No lithium exposure | **1.28** | **1.07–1.52** | 1.13 | 0.95–1.35 | 1.08 | 0.91–1.28 | 0.99 | 0.83–1.17 | 0.86 | 0.73–1.02 |
| Sustained lithium exposure Vs No lithium exposure | **0.47** | **0.37–0.6** | **0.52** | **0.4–0.67** | **0.49** | **0.38–0.65** | **0.58** | **0.44–0.77** | 0.84 | 0.6–1.18 |
| Mean age | 1 | 1.0–1.0 | 1 | 1.0–1.0 | 1 | 1.0–1.0 | 1 | 1.0–1.0 | 1 | 1.0–1.0 |
| Sex (females Vs males) | **0.65** | **0.56–0.75** | **0.66** | **0.57–0.77** | **0.66** | **0.57–0.77** | **0.66** | **0.57–0.77** | 0.67 | 0.58–0.78 |
| 18,001 - 100,000 € Vs < 18,000 € | **0.74** | **0.61–0.91** | **0.73** | **0.6–0.9** | **0.73** | **0.6–0.9** | **0.73** | **0.6–0.89** | 0.73 | 0.59–0.89 |
| > 100,000 € Vs < 18,000 € | 1.53 | 0.57–4.09 | 1.63 | 0.61–4.36 | 1.67 | 0.62–4.49 | 1.64 | 0.61–4.39 | 1.65 | 0.62–4.42 |
| BD-II Vs BD-I | 0.83 | 0.66–1.03 | 0.85 | 0.68–1.06 | 0.85 | 0.68–1.06 | 0.85 | 0.68–1.07 | 0.86 | 0.68–1.07 |
| BD-NOS Vs BD-I | 0.46 | 0.15–1.42 | 0.47 | 0.15–1.48 | 0.47 | 0.15–1.45 | 0.47 | 0.15–1.47 | 0.49 | 0.16–1.52 |
| Combination with antidepressants (yes Vs no) | 0.84 | 0.7–1.0 | 0.86 | 0.72–1.03 | 0.86 | 0.72–1.03 | 0.87 | 0.73–1.05 | 0.89 | 0.74–1.07 |
| Combination with antipsychotics (yes Vs no) | **1.56** | **1.24–1.97** | **1.62** | **1.28–2.05** | **1.64** | **1.29–2.07** | **1.64** | **1.3–2.07** | 1.63 | 1.29–2.07 |
| Combination with mood stabilizers (yes Vs no) | 0.88 | 0.73–1.06 | 0.9 | 0.75–1.09 | 0.92 | 0.76–1.11 | 0.93 | 0.77–1.12 | 0.94 | 0.78–1.14 |
| Comorbidities (1 Vs 0) | **3.49** | **2.68–4.54** | **3.4** | **2.6–4.43** | **3.37** | **2.59–4.4** | **3.38** | **2.59–4.4** | 3.36 | 2.58–4.38 |
| Comorbidities (≥2 Vs 0) | **10.6** | **8.48–13.24** | **10.54** | **8.42–13.2** | **10.47** | **8.36–13.11** | **10.61** | **8.47–13.28** | 10.83 | 8.65–13.56 |

This table presents hazard ratios (HR) and 95% confidence intervals (CI) from Cox proportional hazards regression models assessing the association between lithium use patterns and all-cause mortality at different DDD thresholds. Lithium non-users serve as the reference group. Significant results are reported in bold

# Appendix 5. Results from the Cox proportional hazards model assessing the interaction between lithium use patterns and medical comorbidities.

| **Variable** | **HR** | **95% CI** | **p-value** |
| --- | --- | --- | --- |
| Partial or intermittent lithium exposure Vs No lithium exposure | **1.86** | **1.20–2.90** | **0.005** |
| Sustained lithium exposure Vs No lithium exposure | 1.21 | 0.65–2.25 | 0.543 |
| Comorbidities (1 Vs 0) | **4.39** | **3.04–6.34** | **<0.001** |
| Comorbidities (≥2 Vs 0) | **15.27** | **11.13–20.94** | **<0.001** |
| Mean age | 1 | 0.996–1.003 | 0.825 |
| Sex (females Vs males) | **0.67** | **0.57–0.77** | **<0.001** |
| 18,001 - 100,000 € Vs < 18,000 € | **0.73** | **0.59–0.89** | **0.002** |
| > 100,000 € Vs < 18,000 € | 1.65 | 0.62–4.42 | 0.319 |
| BD-II Vs BD-I | 0.85 | 0.68–1.07 | 0.172 |
| BD-NOS Vs BD-I | 0.48 | 0.15–1.48 | 0.199 |
| Combination with antidepressants (yes Vs no) | 0.88 | 0.73–1.05 | 0.166 |
| Combination with antipsychotics (yes Vs no) | **1.61** | **1.27–2.04** | **<0.001** |
| Combination with mood stabilizers (yes Vs no) | 0.94 | 0.78–1.13 | 0.515 |
| Discontinuous lithium use × Comorbidities (1) | **0.55** | **0.31–0.98** | **0.043** |
| Continuous lithium use × Comorbidities (1) | 0.57 | 0.24–1.34 | 0.196 |
| Discontinuous lithium use × Comorbidities (≥2) | **0.41** | **0.25–0.67** | **<0.001** |
| Continuous lithium use × Comorbidities (≥2) | 0.49 | 0.23–1.03 | 0.06 |

This model explores whether the association between lithium use and all-cause mortality varies according to the burden of medical comorbidities. It includes interaction terms between lithium use (continuous or discontinuous vs. non-use) and number of comorbidities (1 or ≥2 vs. none), adjusting for demographic and clinical covariates. The model demonstrated good discriminatory capacity (concordance = 0.787, SE = 0.008), with highly significant overall fit (Likelihood ratio test = 803.5 on 17 df; Wald test = 598.2 on 17 df; log-rank test = 883 on 17 df; all p < 0.001).

Significant results are reported in bold.
